# Antibiotic free selection for the high level biosynthesis of a silk-elastin-like protein

**DOI:** 10.1038/srep39329

**Published:** 2016-12-16

**Authors:** Mário Barroca, Paulo Rodrigues, Rómulo Sobral, M. Manuela R. Costa, Susana R. Chaves, Raul Machado, Margarida Casal, Tony Collins

**Affiliations:** 1 Centre of Molecular and Environmental Biology (CBMA), Department of Biology, University of Minho, Campus de Gualtar, 4710-057 Braga, Portugal; 2 Biosystems and Integrative Sciences Institute (BioISI), Plant Functional Biology Centre, University of Minho, Campus de Gualtar, 4710-057 Braga, Portugal

## Abstract

Silk-elastin-like proteins (SELPs) are a family of genetically engineered recombinant protein polymers exhibiting mechanical and biological properties suited for a wide range of applications in the biomedicine and materials fields. They are being explored as the next generation of biomaterials but low productivities and use of antibiotics during production undermine their economic viability and safety. We have developed an industrially relevant, scalable, fed-batch process for the high level production of a novel SELP in *E. coli* in which the commonly used antibiotic selection marker of the expression vector is exchanged for a post segregational suicide system, the separate-component-stabilisation system (SCS). SCS significantly augments SELP productivity but also enhances the product safety profile and reduces process costs by eliminating the use of antibiotics. Plasmid content increased following induction but no significant differences in plasmid levels were discerned when using SCS or the antibiotic selection markers under the controlled fed-batch conditions employed. It is suggested that the absence of competing plasmid-free cells improves host cell viability and enables increased productivity with SCS. With the process developed, 12.8 g L^−1^ purified SELP was obtained, this is the highest SELP productivity reported to date and clearly demonstrates the commercial viability of these promising polymers.

Materials and biomaterials based on proteins have attracted much attention in recent years due to their unique mechanical and biological properties^1^,^2^,^3^,^4^ which rival those of synthetic polymers and lend them to numerous applications in a wide range of areas^5^,^6^,^7^,^8^,^9^,^10^,^11^. These protein based polymers (PBPs) are expected to substitute synthetic polymers in many applications as they offer the advantages of being monodisperse, biodegradable, biocompatible and more environmentally friendly^1^,^2^,^3^. Furthermore, the genetically encoded template-based synthesis of these polymers provides for precise control of their design and the fabrication of novel customised biopolymers tailored for a particular application. Some potential applications include drug delivery, tissue engineering and as constituents of implanted medical devices, as well as surgical applications such as soft tissue augmentation and bone repair^5^,^6^,^7^,^8^,^9^,^10^,^11^.

Most commonly, PBPs are inspired by naturally occurring fibrous proteins such as silk, elastin and collagen and make use of the repetitive amino acid sequence motifs characteristic of these proteins and responsible for their favourable mechanical properties^1^,^2^. We have synthesised a number of novel artificial block copolymers combining the repetitive amino acid sequence motifs of silk and elastin^12^. These silk-elastin-like proteins (SELPs) combine the crystallinity and mechanical strength of silk with the high resilience and water solubility of elastin in a single structure. They have been successfully processed into hydrogels, fibers and films and shown to be suitable for application as biomaterials in tissue engineering, namely as wound dressing for skin regeneration^13^ and in ophthalmic applications^14^, but also in general materials science as transparent insulating material for photonics and electronics^14^. In addition, SELPs are also promising candidates for use in drug and gene delivery, tissue culture and as biodegradable plastics^1^,^2^,^15^.

The widespread application of SELPs and PBPs is currently limited by their low production levels^16^,^17^,^18^,^19^,^20^ and hence high production costs, with the highest SELP productivity reported prior to initiation of our studies being only 85 mg per litre of production culture^20^. In an attempt to enhance the commercial viability of these novel polymers we undertook a project aimed at increasing SELP productivity and developing a scalable cost effective production process for these^21^,^22^. Our initial study focused on optimising batch production in the *E. coli* BL21(DE3)-pET expression system via an in-depth empirical approach comparing all process variables and enabled an increase in productivity up to 500 mg/L purified SELP^21^. In addition, factors potentially limiting further improved productivities were identified and included an accumulation of the metabolic byproduct acetate to toxic levels, plasmid instability of culturable cells and increased host cell metabolic burden following induction of production^21^. Subsequently, we circumvented the former limitation by developing and optimising a scalable fed-batch process which reduced acetate accumulation and increased SELP yield to 4.3 g L^−1^, the highest reported value for any PBP to date^22^. In the present study, we attempted to address the latter limitations identified and further increase SELP productivity.

Our previous studies have shown that ampicillin resistance as a selective marker for plasmid stabilisation during high cell density production of proteins is ineffective due to the rapid degradation of ampicillin by the antibiotic resistance protein β-lactamase^21^. This ineffectiveness of ampicillin, in addition to the deprecation by regulatory authorities of its use in biotherapeutics and biomaterials production due to safety concerns^23^,^24^,^25^, prompted us to investigate other plasmid stabilisation systems. Indeed, use of β-lactam antibiotics such as ampicillin poses safety risks associated with their potential for causing serious hypersensitivity reactions in patients and for leading to the spread of antibiotic resistance traits to the environments^25^,^26^. Furthermore, their use, and the necessity for additional purification and testing to ensure their absence in the final product leads to higher process costs. The antibiotic kanamycin is more readily accepted^25^ but a recently developed antibiotic free system, the separate-component-stabilisation system (SCS)^26^,^27^, offers a means of avoiding the use of costly antibiotics and potentially improving both plasmid segregational stability and product yield and safety. This post-segregational stabilisation system employs a toxin producing host which kills host cells that have lost the plasmid and an anti-toxin producing expression vector which inactivates the toxin, thereby ensuring persistence of plasmid-containing cells only. In addition, as compared to antibiotic resistance markers, SCS has an improved safety profile as it is a naturally occurring system innocuous to eukaryotic cells^26^. Furthermore, it is expected to impose a reduced metabolic burden on host cells due to the small sizes of the toxin (100 amino acids) and anti-toxin (90 amino acids) as well as the regulated expression of these (toxin production only in the absence of anti-toxin)^25^,^26^,^27^. In contrast, the relatively large antibiotic-resistance proteins (β-lactamase: 263 amino acids + 23 amino acid signal sequence; aminoglycoside 3′-phosphotransferase: 271 amino acids) are constitutively expressed from the pET expression vectors and indeed have already been shown to have a deleterious effect on host cells^24^,^28^,^29^.

In this study, ampicillin and kanamycin resistance markers, as well as the SCS system and a plasmid construct devoid of any antibiotic resistance marker were compared for the fed-batch production of a SELP and the production process optimised for improved product yield. In addition, a detailed comparative characterisation of the processes and of the effects on SELP productivity and host cell metabolism, physiology and integrity was carried out and allowed for a better understanding of the impact and operation of the plasmid stabilisation systems examined.

## Results

Plasmid instability negatively impacts product yield and process economics and is a major concern in the industrial production of proteins. In our earlier studies optimising the production of a novel SELP (SELP-59-A), a rapid reduction in the proportion of culturable cells containing plasmid was observed following induction of production^21^,^22^. In the present study we attempted to investigate this further by comparing the use of three different plasmid stabilisation systems in the fed-batch production of SELP-59-A: ampicillin resistance (Amp^R^), kanamycin resistance (Kan^R^) and the Separate Component Stabilisation System (SCS + Amp^R^). In addition, we investigated the effect of removing the antibiotic resistance gene from the SCS expression vector (SCS) to examine if the resultant reduced plasmid size and reduced metabolic burden would affect process performance.

### SCS as a Tool for Improving SELP Productivity

A modified, previously developed fed-batch process with a shortened pre-induction phase (induction at a dry cell weight of 25 g L^−1^) and prolonged post-induction phase (8 h) was employed for the high cell density production of SELP-59-A with each of the 4 constructs prepared. 5 ± 0.6 g L^−1^ purified SELP was obtained with both SCS constructs (SCS + Amp^R^ and SCS) under the conditions used, whereas 3.5 ± 0.5 g L^−1^ was obtained with the Amp^R^ construct and 3.4 ± 0.5 g L^−1^ with the Kan^R^ construct. This represents a ~40% increase for the SCS system and clearly demonstrates the effectiveness of this in increasing recombinant protein yield.

### Comparing Process Performance

**Figure 1:**
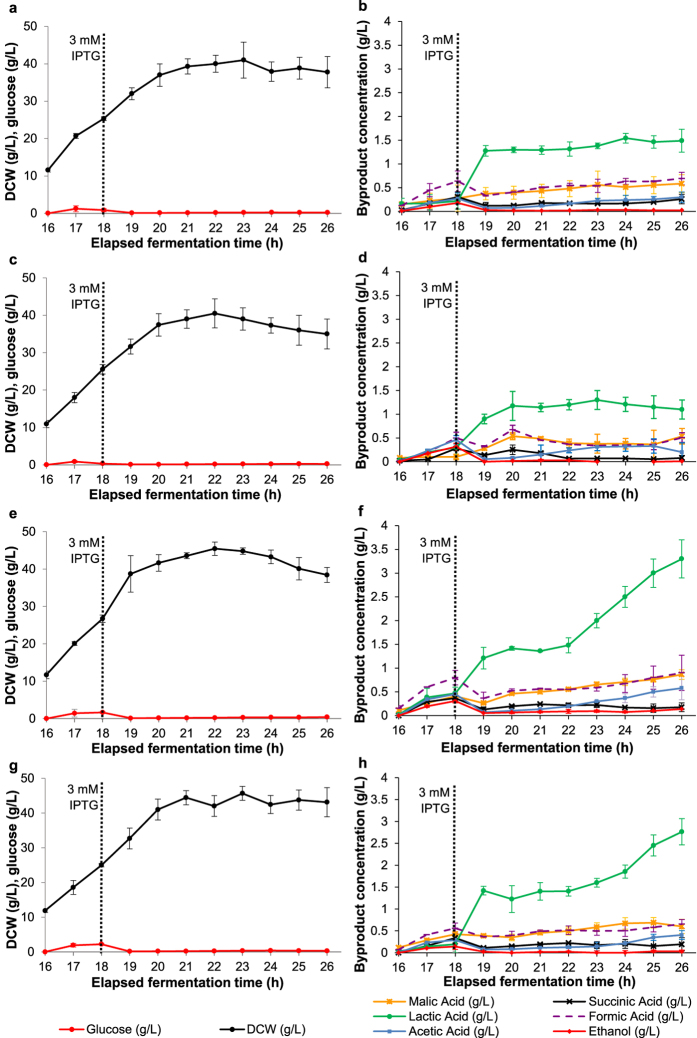
Monitoring of process parameters during the fed-batch phase of production. Results for the dry cell weight (DCW) and glucose concentrations as a function of time (**a,c,e** and **g**) and the variation in organic acids and ethanol levels as monitored by HPLC (**b,d,f** and **h**) are shown for the Amp^R^ (**a** and **b**), Kan^R^ (**c** and **d**), SCS + Amp^R^ (**e** and **f**) and SCS (**g** and **h**) constructs. The vertical dotted line indicates the time point of IPTG induction. Induction was carried out at a dry cell weight of 25 g L^−1^ with 3 mM IPTG for 8 hours. Error bars display the standard deviation of three repeat productions.

For all four constructs examined, similar biomass production and glucose accumulation profiles were observed and, with the exception of lactic acid, highly similar metabolic byproduct accumulation profiles were also noted (Fig. 1). Dry cell weight (DCW) increased during fed-batch feeding and initially also following induction (for ~3 h) but then eventually decreased, probably resultant of the increased metabolic stress imposed on host cells. This is believed to be due to a deviation of cell resources from biomass production to recombinant protein production and is commonly reported with *E. coli* as an expression host^21^,^22^,^30^,^31^. Glucose, malic acid, succinic acid, formic acid, acetic acid and ethanol were maintained at low concentrations (<1 g L^−1^) during the process but, as previously reported^22^, lactic acid drastically increased following induction (Fig. 1b). Lactic acid concentrations up to approximately 1 to 1.5 g L^−1^ for cultures with the Amp^R^ and Kan^R^ constructs were observed but use of the SCS constructs resulted in higher concentrations, averaging around 3 g L^−1^. As previously discussed^22^, this increase in lactic acid production following induction is probably resultant of a diversion of cell resources during SELP production. Therefore, the higher lactic acid levels observed with the SCS constructs are probably related to the higher SELP productivity with these constructs and hence greater pathway deregulation. Finally, the similar results observed with both SCS constructs, in the presence and absence of the ampicillin selection marker gene, indicate that the resistance marker has little effect on the host under the conditions used here.

### Plasmid Stability Versus Host Cell Culturability

**Figure 2:**
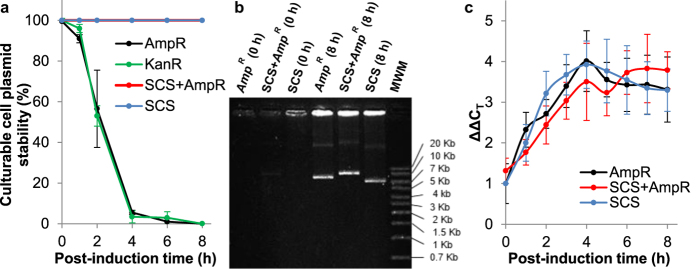
Plasmid stability following induction. The comparative analysis of plasmid stability/content by culturable cell counts (**a**) agarose gel analysis (**b**) and RT-qPCR (**c**) are shown for the fed-batch productions with the Amp^R^, Kan^R^, SCS + Amp^R^ and SCS constructs. The culturable cell count displays the number of cells forming colonies on antibiotic-containing media as a percentage of the total number of culturable cells. Note that SCS and SCS + Amp^R^ gave the same results and hence overlap in (**a**). The agarose gel analysis provides a visual comparison of the intracellular plasmid content before and following 8 hours induction. qPCR results are expressed as the fold ratio of the normalized plasmid amount. Error bars display the standard deviation of three repeat productions.

For monitoring of plasmid segregational stability, initially, the most commonly used method was employed in which the number of culturable cells on antibiotic-containing media is calculated as a percentage of that on antibiotic-free media (Fig. 2a). Hence only the productions with the Amp^R^, Kan^R^ and SCS + Amp^R^ vectors could be investigated.

From Fig. 2a it can be seen that, as previously reported^21^,^22^, use of the ampicillin selection vector resulted in a rapid reduction in the proportion of plasmid-containing culturable cells following induction. 100% of culturable cells contained plasmid before induction and already at 6 hours post induction only plasmid free cells were detected under the conditions used. Similar results were observed with the Kan^R^ marker (Fig. 2a). However, with the SCS system all culturable cells were found to contain plasmid under the conditions used, even after 8 hours of induction. This suggests higher plasmid content for the fermentations with the SCS system. However, and in contrast to this, the comparative quantification of the intracellular plasmid content in the bioreactor by both agarose gel visualisation (Fig. 2b) and quantitative PCR (qPCR, Fig. 2c) indicated no significant differences between the various constructs. For the latter (Fig. 2c), it can be seen that plasmid content increased during the first ~3 to 4 hours and then levelled off at the higher levels attained with all constructs and with no significant difference being observed. Similarly, for the agarose gel study, bands are poorly detectable under the conditions used before induction, but plasmid content increases following induction to higher comparable levels with all constructs, thereby further demonstrating that no significant differences in plasmid content were detected with the techniques used in this study.

**Figure 3:**
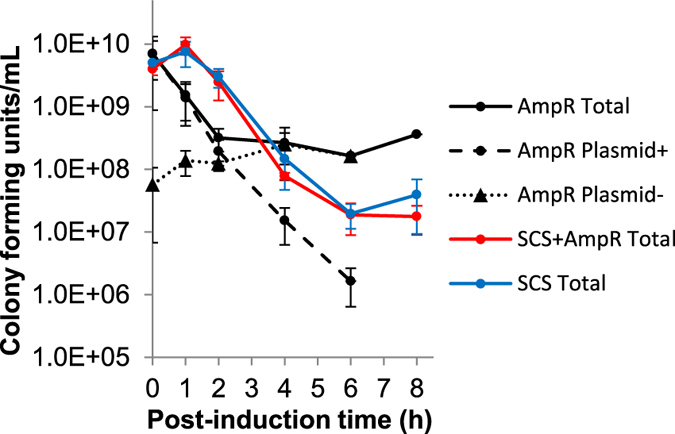
Culturable cell numbers following induction. The total number of colony forming units (enumerated on LB media) for the fed-batch production with the Amp^R^ (black), SCS + Amp^R^ (red) and SCS (blue) constructs are shown. The numbers of plasmid-bearing cells (enumerated on LB + Amp media) and those calculated for plasmid-free cells are shown for the Amp^R^ construct only. All culturable cells for the SCS + Amp^R^ and SCS constructs contained plasmid, no plasmid-free cells were detected, hence the total cell numbers shown here also represent the number of plasmid-containing cells for these. Error bars display the standard deviations for three repeat productions.

**Figure 4:**
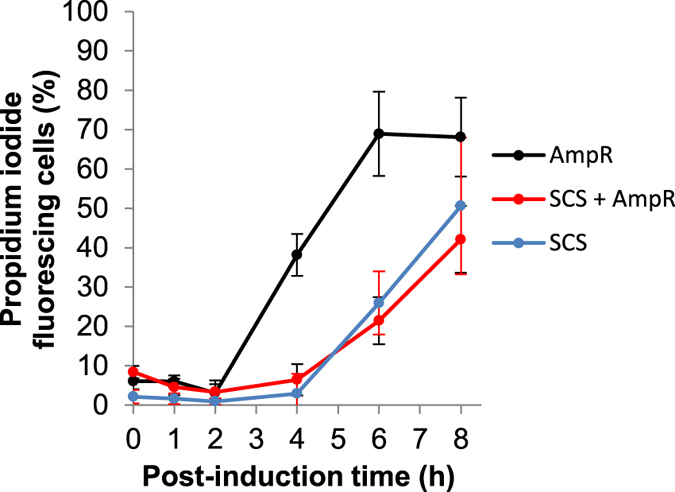
Flow cytometry analysis of host cell membrane permeability. The % of propidium iodide stained cells for the Amp^R^ (black), SCS + Amp^R^ (red) and SCS (blue) constructs are shown as a function of the post-induction time. Error bars display the standard deviations for three repeat productions.

Figure 3 shows the results for the culturable cell counts with the Amp^R^, SCS + Amp^R^ and SCS constructs as well as the calculated number of plasmid-bearing and plasmid-free cells with the Amp^R^ marker. No plasmid-free cells were observed for the SCS constructs but this is not unexpected with the protocol used, as these, even if present in the bioreactor culture medium would be prevented from growing on the LB plate medium due to toxin production from the host chromosome. Furthermore, while an initial increase in cell density was observed following induction in all cases (Fig. 1), in contrast, here (Fig. 3) it can be seen that a very rapid decline in culturable cell numbers immediately occurs with the Amp^R^ construct. The number of culturable plasmid-containing cells drastically decreased throughout the experiment but the total (plasmid-free + plasmid-containing) culturable cell number eventually levelled off due to the growth of plasmid-free cells. With the SCS system, an initial increase during the first hour following induction was indeed observed for the total cell count, which is 100% plasmid containing, but this was then also followed by a rapid decrease in cell numbers. These discrepancies, taken together with Fig. 2b and c where similar plasmid content is noted, suggests that the culturable cell counts (Fig. 3) and related culturable cell plasmid stability measurements (Fig. 2a) used here are not a true measure of plasmid stability. They do not simply indicate, as originally suggested, increased plasmid content with the SCS construct, but are more a measure of cell culturability or colony forming ability, indicating a more rapid loss of cell viability and culturability when using the Amp^R^ construct. Indeed, in agreement with this, our flow cytometry assessment of cell viability using propidium iodide (PI) as probe (Fig. 4) indicated a more rapid loss of membrane integrity for cells with the Amp^R^ construct. Here, live cells with intact membranes exclude the PI dye whereas it easily penetrates dead or damaged cells and interacts with cell DNA leading to diffuse red fluorescence when excited with blue light at 488 nm. It can be seen from Fig. 4 that the onset of the PI-DNA interaction and fluorescence occurs earlier for cultures with the Amp^R^ construct than for both SCS constructs and hence indicates a more rapid accumulation of compromised cells for the former.

**Figure 5:**
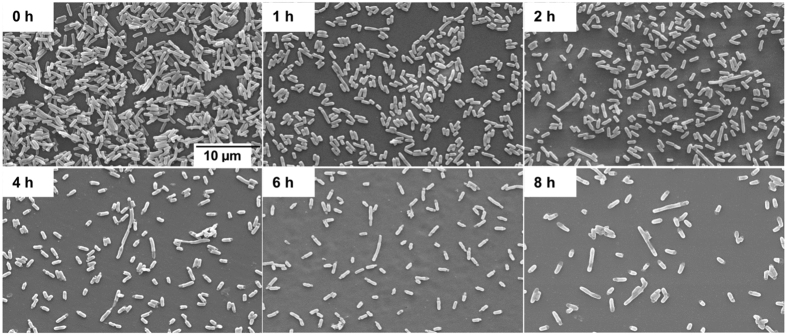
Scanning electron micrographs of *E. coli* host cells at 0, 1, 2, 4, 6 and 8 hours post-induction. Only the results for the fed-batch productions with SCS + Amp^R^ are shown, no significant differences were seen with any of the other constructs studied. Images were acquired at a magnification of 3000×.

Cell morphology was also studied but no significant differences were observed with the various constructs (Fig. 5). At the time of induction, the majority of cells show typical *E. coli* size and shape, but with increasing induction time an increasing number of cells with ‘pinched’ and transparent terminals as well as cell fragmentation and an increase in the proportion of elongated cells is seen under the conditions used. Such changes in cell structure are indicative of cell stress and have been previously reported during recombinant protein production in *E. coli*^32^.

### Maximising SELP-59-A Productivity

Having shown that both SCS constructs studied allowed for similarly improved SELP productivity, construct SCS was chosen for further studies to determine the maximum achievable SELP productivity due to the absence of the antibiotic resistance marker on its backbone. This construct was used with the previously optimised fed-batch process wherein the DCW on induction was increased to 75 g L^−1^ (the limit achievable with the bioreactor system used) but here, as a result of the improved post induction viability of cells with this construct, longer post induction times were investigated.

**Figure 6:**
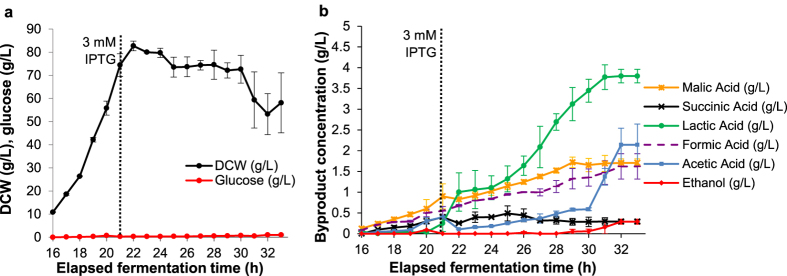
Process parameters for the fed-batch production process with the SCS vector construct. Results for the dry cell weight (DCW) and glucose concentrations as a function of time (**a**) and the variations in organic acid and ethanol concentrations as monitored by HPLC (**b**) are shown for the 12 hour post-induction period. The vertical dotted line indicates the time point of IPTG induction. Induction was carried out at a dry cell weight of 75 g L^−1^ with 1 mM IPTG. Error bars display the standard deviation of three repeat productions.

From Fig. 6 it can be seen that following induction DCW initially increased before levelling off and then decreasing. Glucose concentrations were maintained at low levels throughout the duration of the experiment but organic acids increased, with in particular lactic acid, but also malic and formic acid increasing significantly. Furthermore, the concentration of acetic acid, and to a lesser extent ethanol, suddenly increased 10 hours post-induction, which concomitant with a reduction of DCW is indicative of cell disruption. A strong increase in culture viscosity, probably due to a release of chromosomal DNA, and the release of proteins to the extracellular environment as observed by SDS analysis supports this. Reduced SELP yields were hence observed with the 12 h post-induction period (9.3 ± 2 g L^−1^) and the highest production was observed 8 hours post-induction (12.8 ± 2 g L^−1^), with lower amounts being obtained for the shorter 4 hour post induction time (6.8 ± 1 g L^−1^).

## Discussion

In this study, it is shown that use of the SCS system for the fed-batch production of SELP-59-A in *E. coli* significantly enhances productivity as compared to the use of antibiotic selection markers. With the optimised process developed herein 12.8 g L^−1^ purified SELP was obtained and represents the highest reported SELP productivity to date, being 3-fold higher than that obtained with our previously optimised fed-batch process^22^ and 150-fold higher than that reported by other groups^20^. Indeed, this SCS system offers many advantages for the fed-batch production of recombinant proteins in *E. coli*. It not only improves product yield, but also improves the safety profile by eliminating the use of antibiotics and antibiotic resistance markers and reduces costs by negating the need for antibiotic addition and supplementary purification and testing to ensure product safety. It is suitable for the GMP production of proteins and biotherapeutics such as PBPs and its use here in the scalable fed-batch process demonstrates its potential in industrially relevant processes. Furthermore, its use in the present study for the high level production of a novel SELP demonstrates the commercial viability of SELPs and PBPs in general and should allow for a more widespread acceptance of these.

No significant differences in process performance or host cell physiology were noted when the SCS system was used in the presence or absence of an antibiotic selection marker. Interestingly, previous studies have indicated that constitutive expression of antibiotic resistance markers negatively affects host cells and in fact marker accumulation up to levels as high as 20% of total cellular protein^29^ and even inclusion body formation have been reported^23^. This imposes a metabolic load on host cells and it has been shown that its elimination can improve process performance, namely for plasmid production processes^23^. However, protein overproduction imposes a severe metabolic burden on host cells^30^,^31^, as was seen here for SELP overproduction, and it is possible that the negative effects resultant from the β-lactamase are negligible as compared to the effects imposed by this overproduction^33^ and hence are masked by these.

Our studies investigating plasmid stability using the traditional approach of enumerating the number of plasmid-containing colony-forming cells as a proportion of the total number of culturable cells indicated an apparent increased stability with the SCS system (Fig. 2a). Even after 8 hours induction, 100% of the colony-forming units were plasmid-bearing whereas no plasmid-bearing culturable cells were detected 6 hours post induction with the Amp^R^ system under the conditions used. This suggests a higher number of plasmid-containing cells and higher plasmid content for the fermentations with the SCS system which could allow for the higher SELP productivities observed, yet, in contrast, our quantitative studies did not reveal evidence of increased plasmid content. The comparative quantification of the intracellular plasmid content by both agarose gel visualisation (Fig. 2b) and qPCR (Fig. 2c) did not indicate significant differences between the various constructs examined under the conditions used. With all constructs, plasmid content initially increased and then levelled off at similar higher levels. Such an initial increase in plasmid content has in fact been previously reported^34^ and is believed to be due to the decreased cell division following induction being accompanied by continued plasmid replication and hence a resultant higher copy number per cell. Also, while the equivalent plasmid content observed for all constructs (Fig. 2b and c) appears somewhat contradictory to the culturable cell plasmid stability results (Fig. 2a), equal plasmid content under the conditions used may not be unexpected as the SCS system is a post-segregational killer system^27^,^33^,^35^,^36^. It is not a partitioning system that obliges daughter cells to take up a copy of the plasmid during cell division, but rather it prevents the outgrowth and proliferation of plasmid-free cells by toxin inactivation of the host cell DNA gyrase^27^,^33^,^35^,^36^. Thus, in the true sense of the term ‘plasmid stability’, which is a measure of the likelihood with which a plasmid is inherited by daughter cells at cell division, the SCS system would not be expected to enable an enhancement. In the present study, identical fed-batch processes were used with all constructs and hence, as was indeed observed here, the rate of formation of plasmid-free cells and thus also the plasmid content, could be expected to be similar in all cases. Indeed, such results are consistent with a previous study where it was argued that postsegregational killing systems do not increase plasmid stability^36^. Thus, it can be concluded that, under the controlled fed-batch production conditions of our study, with a reduced post-induction feeding rate, equal concentrations of plasmid were observed with all constructs and thereby indicates that something, other than plasmid stabilisation, allowed for the increased SELP production obtained.

In relation to the culturable-cells plasmid stability study, it must be noted that the SCS system prevents outgrowth of plasmid-free cells but with the Amp^R^ construct these can grow and indeed outcompete the slower growing plasmid-containing cells and hence reduce the proportion of these and lead to the results observed (Fig. 2a). That is to say, the relative, but not the absolute level of plasmid-containing cells is reduced with Amp^R^. In addition, as discussed earlier, plasmid-free cells for the SCS system, even if present in the bioreactor, would not be able to form visible colonies and would not be detected with the plating techniques used here for measuring plasmid stability. Therefore, the culturable cell plasmid stability test most commonly used is inappropriate for determining plasmid content in this case, it is not a measure of the number of cells containing plasmid or of the intracellular plasmid content, but a measure of the proportion of dividing culturable cells containing plasmid.

Recombinant protein overexpression places a severe metabolic burden on host cells and its debilitating effects are well known^30^,^31^. It drains resources such as energy, precursor metabolites and protein synthesizing machinery from host cell metabolism and leads to metabolic imbalances, reduced growth rates and cell disruption and death. In the present study, this was manifested by a reduced biomass accumulation (Fig. 1), loss of cell culturability (Fig. 3), loss of membrane integrity (Fig. 4), alteration in cell morphology (Fig. 5) and increased byproduct formation (Figs 1 and 6) as well as, eventually, cell disruption (Fig. 6). Interestingly, while a rapid loss of cell culturability was seen with all constructs following induction (Fig. 3), this loss was more rapid with the Amp^R^ construct, and together with the higher rate of loss of membrane integrity (Fig. 4) indicates a reduced fitness of cells with this construct as compared to those with the SCS system. This apparent stimulating effect of the SCS system on host cells may initially appear somewhat counterintuitive, as the increased SELP production of these would be expected to impact a greater metabolic burden, and indeed the higher lactic acid production of these (Fig. 1) is reflective of an increased metabolic deregulation resultant of this higher SELP production. However, the SCS system prevents growth of plasmid-free cells, whereas with the Amp^R^ construct these can compete with and outgrow the plasmid-bearing cells for nutrients, energy and other resources^27^,^30^,^37^,^38^. This competition could impose an additional stress on the host cells and, consequently, may induce the increased rate of loss of culturability and membrane integrity observed, as well as, most importantly, the reduced SELP productivity with the Amp^R^ system under the conditions used. Thus, hereby, it is suggested that with the controlled fed-batch conditions used here, the SCS system does not enable significantly increased plasmid content as compared to with Amp^R^, but it is an improved cell fitness, due to the absence of competition between plasmid-bearing and plasmid-free cells, that enables improved SELP productivity with this system. It can also be seen that plasmid content, in fact, increases following induction and that the principle limiting factor for improved productivity is the metabolic burden imposed on the host cells as a result of the recombinant protein overexpression. Indeed, this is already well described in the literature^30^,^31^,^33^ and a recent study took successful initial steps in addressing this for the high level production of a recombinant silk protein with a high glycine content by metabolically engineering the host for an increased glycyl-tRNA pool^39^,^40^. SELP is also characterised by a highly repetitive sequence and hence a similar metabolic approach combined with our scalable antibiotic free technique described here may aid in further increasing yields and in better enhancing the economic feasibility of this novel biopolymer.

## Conclusions

Protein based polymers are being explored as the next generation of biomaterials and a critical step in the strategic analysis of their viability is a demonstration of their cost effective production in a format suitable and safe for use in clinical practice. In this study, a scalable, reproducible, industrially relevant production process has been developed for the high level production of a novel SELP with total elimination of the use of antibiotics and antibiotic selection markers. It was shown that use of a post-segregational suicide system, SCS, for the fed-batch production of SELP-59-A in *E. coli* significantly enhanced productivity as compared to the use of antibiotic selection markers. Furthermore, an in-depth characterisation of the process provided insights into the operation of the stabilisation systems and production process and it is suggested that an improved viability of host cells plays a role in the observed increased productivity with SCS.

## Methods

### SELP Polymer

SELP-59-A was used in this study, this consists of a nine times repeated copolymer unit composed of five repeats of the silk amino acid consensus sequence (GAGAGS) and nine repeats of an elastin mimetic sequence (VPAVG)^21^.

### Vector Constructs

All expression vectors used are based on the pET expression vector system (Novagen) and all vector constructs prepared here were confirmed by restriction digestion and agarose gel analyses.

#### SELP-59-A/pET25bAmp^R^

The gene for SELP-59-A had already been inserted in a modified pET25b(+)vector (Novagen) for expression in *E. coli* BL21(DE3)^12^, this vector contains the ampicillin resistance selective marker gene encoding β-lactamase.

#### SELP-59-A/pET29aKan^R^

For the kanamycin resistance marker (neomycin phosphotransferase II), the expression vector pET29a(+) (Novagen) was used. The SELP-59-A gene was excised from SELP-59-A/pET25bAmp^R^ by digestion with *Nde*I and *Blp*I and ligated with T4 DNA ligase into similarly digested pET29a(+). Plasmid constructs were transformed to *E. coli* BL21(DE3) for expression.

#### SELP-59-A/pStaby1.2SCSAmp^R^

For the separate-component-stabilisation system (SCS), the expression vector pStaby1.2 (Delphi Genetics) was used. In addition to SCS, this vector also carries the ampicillin resistance marker, it is a variant of pET21a(+) in which the gene for the antitoxin ccdA has been inserted. The SELP-59-A gene was excised from SELP-59-A/pET25bAmp^R^ by digestion with *Nde*I and *Blp*I and ligated with T4 DNA ligase into similarly digested pStaby1.2. Plasmid constructs were first transformed to the cloning host *E. coli* CYS21 (Delphi Genetics) before transformation to the expression host *E. coli* SE1 (Delphi Genetics), this is a variant of *E. coli* BL21(DE3) with a chromosomal copy of the gene for the ccdB toxin.

#### SELP-59-A/pStaby1.2SCS

The β-lactamase gene and promotor were removed from SELP-59-A/pStaby1.2SCSAmp^R^ by digestion with *Ssp*I and *Eam*1105I, purified fragments were converted to blunt ended fragments by treatment with Accuzyme mix (Bioline) for 30 mins at 72 °C and re-circularised with T4 DNA ligase. Plasmid constructs were transformed to *E. coli* SE1 (Delphi Genetics) for expression.

### Fed-Batch Production

Fed-batch fermentations were performed as previously described^22^ in a BioFlo 110 Modular Benchtop 3 L fermentor (New Brunswick Scientific, NBS). The whole process involved two precultures followed by a production process composed of three phases (batch, fed-batch and induction) in the minimal medium MMLBM as described by Collins *et al*.^22^. The conditions used were as previously detailed^22^, namely: 37 ± 1 °C, pH 6.8 ± 0.1 (25% w/w NH_4_OH and 3 M H_3_PO_4_), minimum dissolved oxygen concentration = 25% (batch phase) and 35% (fed-batch phase) with control by cascading to agitation (200–1100 rpm) and O_2_ feeding (only during the fed-batch phase), gas (air, air + O_2_) flow rate = 5 L min^−1^ and antifoam Y-30 emulsion for foaming control (Sigma). 200 μg/mL ampicillin was used for all precultures and production cultures with the Amp^R^ constructs and 100 μg/mL kanamycin was used for the Kan^R^ construct while no antibiotic was included for the *SELP-59-A/pStaby1.2SCS* construct. All fed-batch studies were repeated at least three times.

For the comparative study of fed-batch productions with the four different vector constructs, a pre-induction growth rate of 0.5 h^−1^, post-induction growth rate of 0.1 h^−1^ and induction at a dry cell weight of 25 g L^−1^ with 3 mM IPTG for 8 hours was used. Here, a higher IPTG concentration (3 mM IPTG) was used than that previously recommended (1 mM IPTG^21^) as this has been shown to have a greater negative effect on the proportion of plasmid containing culturable cells while not affecting the final product yield^21^.

Maximum achievable SELP-59-A productivity was determined for the construct identified as enabling highest plasmid stability by use of the previously optimised fed batch production process^22^ wherein a pre-induction growth rate of 0.4 h^−1^, post-induction growth rate of 0.1 h^−1^ and induction at a dry cell weight of 75 g L^−1^ with 1 mM IPTG was used. The post induction period was optimised by examining productivities and process parameters after 4, 8 and 12 hours post induction incubation.

### Process Monitoring

Process parameters such as glucose, ethanol, organic acids (citric, tartaric, malic, succinic, lactic, acetic and formic), phosphate, nitrogen and biomass concentrations were analysed and compared as previously described^22^.

#### SELP Production Quantification

SELP production levels for the various constructs were determined by weight measurements of purified product as previously described^12^,^22^. A previously developed non-chromatographic approach was used for purification^12^. This involved cell disruption by sonication, overnight acid treatment at pH 3.5 to denature contaminants, centrifugation and SELP precipitation with 20% ammonium sulphate. Following centrifugation, resuspension and dialysis in distilled water, the purified SELP was then lyophilied and weighed.

#### Culturable cell count

Samples were taken at the time of induction and every hour thereafter, serially diluted in sterile phosphate buffered saline (PBS: 8 g L^−1^ NaCl, 0.2 g L^−1^ KCl, 1.44 g L^−1^ Na_2_HPO_4_, 0.24 g L^−1^ KH_2_PO_4_, pH 7.4) and 25 μL of the 10^−3^–10^−7^ dilutions spread plated in triplicate on LB plates (10 g L^−1^ bacto tryptone, 5 g L^−1^ yeast extract, 5 g L^−1^ NaCl, 18 g L^−1^ agar, pH 7) and incubated overnight at 37 °C.

#### Culturable Cell Plasmid Stability

100 colony forming units of the culturable cell count study were repicked to LB + ampicillin (200 μg mL ampicillin) plates and incubated overnight at 37 °C before enumeration.

#### Agarose Gel Electrophoresis for Plasmid Quantification

Plasmid content was evaluated at the time of induction and every two hours thereafter. The OD_600nm_ of culture samples was measured and cell pellets washed twice in PBS before resuspension and dilution in TE buffer (50 mM Tris, 1 mM EDTA, pH 8) to an OD_600nm_ of 2. This was followed by chemical lysis and chromosomal DNA removal with the lysis and precipitation reagents (buffers A1, A2 and A3) of the NucleoSpin Plasmid Kit (Macherey-Nagel) as described by the manufacturer. 400 μL of plasmid extract was concentrated by precipitation with 50% isopropanol overnight at −20 °C, resuspended in 100 μL water and 20 μL loaded on midori green stained 1% agarose gels for visualization and band intensity comparison.

#### qPCR for Relative Plasmid Quantification

Quantitative PCR (qPCR) was used to monitor the relative plasmid content during the fed-batch productions. The OD_600nm_ of culture samples was measured and cell pellets washed twice in PBS before resuspension and dilution in TE buffer to an OD_600nm_ of 1. Following treatment of 100 μL sample with 50 μg/mL RNase A, cells were disrupted by addition of 2% SDS and heating at 75 °C for 5 minutes. Supernatants were diluted 10^4^-fold in water before qPCR analysis. qPCR reactions were performed in duplicate for each sample on a CFX96 Touch™ Real-Time PCR detection system (Bio-rad) with Sso7d fusion polymerase and EvaGreen^®^ dye (SsoFast™ EVAGreen^®^ Supermix, Bio-Rad). Data were analysed with the CFX Manager TMSoftware v3.1 (Bio-Rad). PCR conditions used were: denaturation at 95 °C for 3 minutes followed by 40 cycles of denaturation at 95 °C for 10 s and annealing/extension for 10 s at 63 °C (plasmid) or 60 °C (host chromosome). Amplification specificity and absence of reaction contaminants were confirmed by analysis of melting curves following heating of amplification products from 60 to 95 °C.

For qPCR analysis a variation of the protocol described by Lee *et al*. 2006^41^ was used in which both plasmid and host chromosome content are monitored so as to eliminate sample preparation and processing variations. Primers were designed (GATCCGGCTGCTAACAAAGC; TTAGAGGCCCCAAGGGGTTA) to amplify an 89 bp fragment present on all four vector constructs studied. 0.25 μM primers and an annealing/extension temperature of 63 °C with an amplification efficiency of 102.4% and R^2^ of 0.987 were used. For monitoring of host chromosome content, 0.25 μM primers (ACAAAACGCATATCGACCAGC; GCGATCCTTAACTTTGGTACGC) and an annealing/extension temperature of 60 °C were employed to amplify an 86 bp fragment present on the *E. coli* chromosomal d-1-deoxyxylulose 5-phosphate synthase (dxs) sequence with an amplification efficiency of 103.3% and R^2^ of 0.994. Relative quantification was performed by the ΔΔC_T_ (ΔΔ threshold cycle) method^41^,^42^ and results are expressed as a fold ratio of the normalized plasmid amount.

#### Flow Cytometry

Propidium iodide was used as a probe of membrane integrity with the flow cytometry analysis of samples collected at induction and every two hours thereafter. Culture samples were diluted 10^2^-fold in PBS and stained with 1 μg/mL propidium iodide (Sigma) for 10 minutes in the dark at room temperature. Analysis was performed with an EPICS^®^ XL™ flow cytometer (Beckman Coulter) at a flow rate of 200–500 events per second. The cell population was gated in a histogram of Side Scatter (SS) versus Forward Scatter (fs) and monoparametric detection of propidium iodide fluorescence was performed using FL-3 (488/620 nm). Twenty thousand cells were analysed per sample.

#### Scanning Electron Microscopy

10-fold dilutions of culture samples collected at the time of induction and at 2 hourly intervals until the end of fermentation were analysed by scanning electron microscopy (SEM). Samples were fixed to plastic disc supports by treatment with 2.5% glutaraldehyde in PBS for 1 hour and dehydrated by successive 30 minute immersions in ethanol solutions of increasing concentration (55, 70, 80, 90, 95 and 100% v/v ethanol in water). Following drying at room temperature overnight, samples were coated with a thin gold layer using a Polaron model SC502 sputter coater and visualised on a NanoSEM-FEI Nova 200 scanning electron microscope (Leica Cambridge).

## Additional Information

**How to cite this article**: Barroca, M. *et al*. Antibiotic free selection for the high level biosynthesis of a silk-elastin-like protein. *Sci. Rep.* **6**, 39329; doi: 10.1038/srep39329 (2016).

**Publisher’s note:** Springer Nature remains neutral with regard to jurisdictional claims in published maps and institutional affiliations.
